# Different Roles of a Rat Cortical Thymic Epithelial Cell Line *In Vitro* on Thymocytes and Thymocyte Hybridoma Cells: Phagocytosis, Induction of Apoptosis, Nursing and Growth Promoting Activities

**DOI:** 10.1080/1044667021000003934

**Published:** 2002-06

**Authors:** Dragana Vučević, Miodrag Čolić, Petar Popović, Sonja Gašić

**Affiliations:** Institute of Medical Research MMA. Crnotravska 17 Belgrade 11002 Serbia and Montenegro

## Abstract

In this work, the interaction between a rat cortical thymic epithelial cell (TEC) line (R-TNC.1) with nursing activity and thymocytes as well as BWRT 8 thymocyte hybridoma (TH) cells has been studied. The R-TNC.1 cell line significantly bound thymocytes and TH. Binding was stronger during the first 30 min of cell incubation and was followed by a progressive deadhesion. Among adherent thymocytes the proportion of apoptotic cells increased with culture time which was a consequence of higher capacity of the line for binding of apoptotic than viable cells and induction of apoptosis in a subset of adherent thymocytes. Emperiopolesis activity of this thymic nurse cell (TNC) line was manifested by engulfment of thymocytes as well as TH cells. A subset of viable intra-TNC thymocytes has been triggered to die by apoptosis, whereas other internalized thymocytes have been stimulated to proliferate, as measured by an increase in the percentage of cells in mitosis and higher incorporation of bromodeoxyuridine (BrdU), in comparison to thymocytes cultivated alone. A significant stimulation of proliferation of engulfed TH cells was also observed. The R-TNC.1 cell line efficiently phagocytosed both apoptotic thymocytes and TH, and the process is followed by intra-TNC destruction of ingested cells. Cumulatively, these results suggest different role of the R-TNC.1 clone: phagocytosis of apoptotic cells; induction of apoptotic cell death in a subset of both bound and internalized thymocytes and stimulation of proliferation of a subset of intra-TNC thymocytes or TH cells.


*Developmental Immunology*, 2002 Vol. 9 (2), pp. 63–72

Taylor & Francis healthsciences

### Different Roles of a Rat Cortical Thymic Epithelial Cell Line *In Vitro* on Thymocytes and Thymocyte Hybridoma Cells: Phagocytosis, Induction of Apoptosis, Nursing and Growth Promoting Activities

DRAGANA VUČEVIĆ, MIODRAG ČOLIĆ*, PETAR POPOVIĆ and SONJA GAŠIĆ

*Institute of Medical Research, MMA, Crnotravska 17, 11002 Belgrade, Minor Yugoslavia*

In this work, the interaction between a rat cortical thymic epithelial cell (TEC) line (R-TNC.1) with nursing activity and thymocytes as well as BWRT 8 thymocyte hybridoma (TH) cells has been studied. The R-TNC.1 cell line significantly bound thymocytes and TH. Binding was stronger during the first 30 min of cell incubation and was followed by a progressive deadhesion. Among adherent thymocytes the proportion of apoptotic cells increased with culture time which was a consequence of higher capacity of the line for binding of apoptotic than viable cells and induction of apoptosis in a subset of adherent thymocytes. Emperiopolesis activity of this thymic nurse cell (TNC) line was manifested by engulfment of thymocytes as well as TH cells. A subset of viable intra-TNC thymocytes has been triggered to die by apoptosis, whereas other internalized thymocytes have been stimulated to proliferate, as measured by an increase in the percentage of cells in mitosis and higher incorporation of bromodeoxyuridine (BrdU), in comparison to thymocytes cultivated alone. A significant stimulation of proliferation of engulfed TH cells was also observed. The R-TNC.1 cell line efficiently phagocytosed both apoptotic thymocytes and TH, and the process is followed by intra-TNC destruction of ingested cells. Cumulatively, these results suggest different role of the R-TNC.1 clone: phagocytosis of apoptotic cells; induction of apoptotic cell death in a subset of both bound and internalized thymocytes and stimulation of proliferation of a subset of intra-TNC thymocytes or TH cells.

*Keywords*: Thymic nurse cells; Thymocytes; T cell hybridoma; Adhesion; Proliferation; Apoptosis

### INTRODUCTION

Thymic nurse cells (TNC) are specialized epithelial cells that form unique multi-cellular complexes with thymocytes (Wekerle *et al.*, 1980). Since their first description by Wekerle *et al.* (1980), TNC have been considered as a very intriguing microenvironmental component which may provide an ideal place for T cell differentiation, maturation and education as they express MHC antigens, secrete thymic hormones and cytokines and may present self antigens to very immature thymocytes (Kyewski, 1986; Rieker *et al.*, 1995). In addition, *in vitro* studies have revealed the possible roles of TNC in negative selection either by inducing programmed cell death or simply clearing the thymus of apoptotic cells (Hiramine *et al.*, 1990; 1995; Aguilar *et al.*, 1994).

Over the last years, many debates have been generated about the real status and function of TNC but the precise role of these cells is still unknown. To date, very little is known about the nature and mechanisms of the interaction between TNC and thymocytes. This may be due to the fact that it was difficult to obtain the large number of cells needed for experiments. In the past few years this problem has been resolved by establishing thymic epithelial cell (TEC) lines with nursing activity, isolated from spontaneously growing murine thymic tumors or normal thymus by means of various cloning techniques or immortalization procedures by treating the cells with Simian virus 40 (Nishimura *et al.*, 1990; Ezaki *et al.*, 1991; Li *et al.*, 1992; Philp *et al.*, 1993; Pezzano *et al.*, 1995; 1996).

We previously successfully cloned a TEC line from long-term cultures of the normal AO rat thymus. This line, named R-TNC.1 was characterized as a type of cortical TEC based on its phenotypic and ultrastructural features. It has been demonstrated that the R-TNC.1 cell line possesses nursing activity manifested by the binding and subsequent engulfment of thymocytes or thymocyte

*Corresponding author. Fax: +381-11-662-722. E-mail: vmaimi@eunet.yu

ISSN 1044-6672 print © 2002 Taylor & Francis Ltd
DOI: 10.1080/1044667021000003934

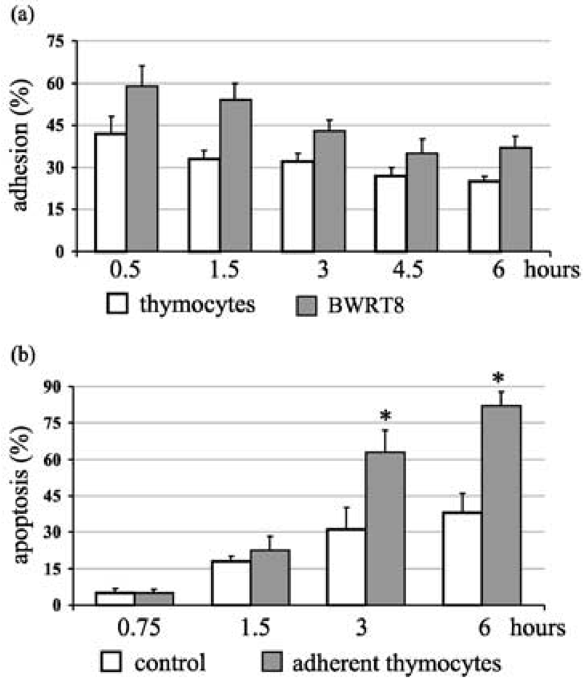

FIGURE 1 (a) Dynamics of thymocyte/TH binding to the R-TNC.1 cell line. Adhesion assay was performed as described in "Materials and methods" section. The percentage of adhesion was determined as ratio between the number of adherent thymocytes/TH and number of total thymocytes/TH added to R-TNC.1 monolayers. Values are mean ± SD of triplicates of one out of three separate experiments. (b) Analysis of apoptotic cells among thymocytes bound to the R-TNC.1 cell line. Results are given as percentage of apoptosis determined by morphological criteria ± SD of triplicates of one out of three separate experiments. $*p < 0.001$ compared to corresponding controls (thymocytes cultivated in medium without TEC).

hybridoma (TH) cells. The line binds double positive $CD4^+CD8^+$ thymocytes with low expression of αβTCR and phenotypicaly different TH. In these interactions a number of adhesion molecules (CD2, CD4, CD8, LFA-1, ICAM-1 and Thy1) were involved (Čolić *et al.*, 1994; 1997; 1998).

Using an *in vitro* coculture system, we studied in this work the fate of thymocytes and BWRT 8 TH ($CD4^{hi}CD8^{lo}\alpha\beta TCR^{hi}$) after their interaction with R-TNC.1 cells. We found that the TEC line binds and engulfs viable and apoptotic thymocytes and TH cells, induces apoptosis of both adherent and internalized thymocytes and stimulates proliferation of a subset of intra-TNC thymocytes and TH. Certain specificities of this first rat TNC line described so far have been discussed.

### RESULTS AND DISCUSSION

### R-TNC.1 Cell Line Binds both Viable and Apoptotic Thymocytes

We have previously demonstrated that a rat cortical TEC line (R-TNC.1) forms complexes with autologous thymocytes *in vitro* (Čolić *et al.*, 1994). In this work, we first studied the dynamics of the adhesion process. As shown in Fig. 1a after an initial rapid binding which was maximal 30 min following the cell contact (42 ± 6% adherent thymocytes) adhesion gradually decreased up to 6 h (25 ± 2%). Similar kinetics were observed when a TH (BWRT 8) was used (Fig. 1a). However, binding of TH cells was stronger in comparison to thymocytes. Hirata *et al.* (1991) also found that the number of thymocytes adhering to the mouse TEL-2 line with nursing activity was greatest at 1 h, followed by their significant detachment after prolonged cocultivation. We have previously demonstrated that LFA-1 and its ligand ICAM-1 are partly involved in the binding of thymocytes to both cortical, R-TNC.1 and medullary (TE-R2.5) cell lines in the early adhesion phase (30 min) (Čolić *et al.*, 1994; 1997). After prolonged incubation (3 h) inhibitory effects of these mAbs were not observed (Čolić *et al.*, 1994; 1997 and our unpublished data). These results are in agreement with those of Lepesant *et al.* (1990) who showed that LFA-1 is involved in stabilization of the early rapid phase of thymocyte adhesion to a murine TEC line which constitutively expressed ICAM-1 in culture.

Uncompleted blocking of thymocyte adhesion to R-TNC.1 cells by anti LFA-1 and anti-ICAM-1 mAbs suggests that other adhesion molecules are involved in these processes, as suggested by many authors (Li *et al.*, 1992; Villa-Verde *et al.*, 1994; 1995; Čolić *et al.*, 1994; 1997; Oliveira-dos-Santos *et al.*, 1998). We previously identified that CD4 and Thy 1 participate in the early phase of TEC/thymocyte binding (Čolić *et al.*, 1994) but their involvement in the late adhesion phases has not been studied.

We next analyzed whether R-TNC.1 cells bind viable or apoptotic thymocytes. Results given in Fig. 1b shows that both viable and apoptotic cells were seen in the population of adherent thymocytes. The proportion of apoptotic adherent thymocytes increased with culture time as did in the population of whole thymocytes cultivated alone (spontaneous apoptosis) (Fig. 1b). After 3 and 6 h, respectively, the differences were statistically very significant ($p < 0.001$). Among adherent TH all cells were viable (data not shown). Mukamoto *et al.* (1999) showed that 60–70% of thymocytes attached to chicken thymic stromal cells (TSC). They also found that a subset of thymocytes in the contact with TSC undergoes apoptotic death. However, the authors did not check another possibility that TSC line binds apoptotic thymocytes spontaneously died in culture. Therefore in our further experiments we tested whether the R-TNC.1 cell line preferentially binds apoptotic thymocytes and/or induces apoptosis of adherent thymocytes.

### R-TNC.1 Cell Line Preferentially Binds Apoptotic Thymocytes

To answer the question whether apoptotic thymocytes better adhere to R-TNC.1 cells than viable ones, we

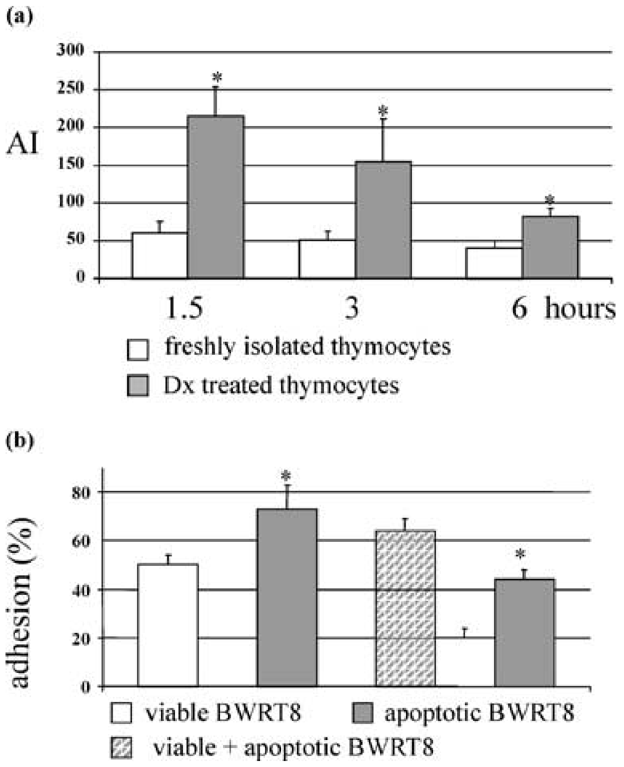

FIGURE 2 (a) Comparison of binding dynamics of freshly isolated and Dx treated thymocytes to R-TNC.1 cell line. Thymocytes were incubated with Dx (100 nM) at 37°C for 24 h before using for the adhesion assay on multi spot glass slides. Values are given as adhesion index (AI). AI = Number of bound thymocytes (total, viable or apoptotic)/100 R-TNC.1 cells. The levels of apoptosis in control, freshly isolated thymocytes were: 0 h (2%), 1.5 h (19%), 3 h (29%) and 6 h (38%). Apoptosis in Dx treated thymocytes were higher than 98% in all time points studied. Values (mean ± SD) of non-aplicates of one representative experiment (out of three ones with similar results) are given. All differences are statistically significant ($*p < 0.001$). (b) Adhesion of viable and apoptotic BWRT 8 TH to the R-TNC.1 cell line. TH apoptosis (higher than 98%) was induced by prolonged cultivation (for 72 h) at higher density ($5 \times 10^5$ TH/ml). Viable, apoptotic or a mixture of equal proportion of viable and apoptotic TH were added to the confluent monolayers of R-TNC.1 cells in 96-well plates and incubated 30 min at 37°C. The percentage of TH adhesion was determined as ratio between the number of adherent TH and the number of total TH added to TEC monolayers. Values are given as mean ± SD of triplicates of one representative experiment. $*p < 0.05$ compared to viable cells.

compared binding of apoptotic, dexamethasone (Dx) treated thymocytes with control (freshly isolated) thymocytes in our adhesion assay. Results presented in Fig. 2a show that binding of Dx treated thymocytes to the R-TNC.1 cell line was much higher in all time points studied. Similar results were confirmed when apoptotic BWRT 8 cells were used. When a mixture of equal proportion of viable and apoptotic TH were cultivated with R-TNC.1 cells, higher percentages of apoptotic cells adhere to the line ($p < 0.05$, compared to viable cells) (Fig. 2b). Cumulatively these results suggest that the R-TNC.1 cell line preferentially binds apoptotic thymocytes (hybridoma cells).

Neither study concerns to the TEC/thymocyte adhesion elaborated similar phenomenon. There are several explanations of our results. At first it might be a consequence of non-specific attachment of apoptotic cells to TEC. This is in agreement with our unpublished data that apoptotic thymocytes also better adhere to the fibroblast line (L929) than viable cells, but contradicts with our results using thymic macrophages (data not shown). Alternatively, better attachment of apoptotic cells to the TEC line could be due to the expression of specific receptors on this TEC line for ligands present on apoptotic cells. It is also possible that the affinity of certain adhesion molecules that are present on viable thymocytes increased on cells undergoing apoptosis, as shown for some other cell-surface molecules (Kishimoto *et al.*, 1995).

### R-TNC.1 Cell Line Induces Apoptosis of Adherent Thymocytes

To study whether R-TNC.1 cells also induce apoptosis of thymocytes we first examined is there any difference in survival of thymocytes cultivated with TEC in comparison to survival of thymocytes cultivated alone. Results presented in Fig. 3a shows that survival of thymocytes in coculture with R-TNC.1 cells was statistically lower in comparison to the values of corresponding control (thymocytes without TEC), indicating that the R-TNC.1 cell line might induce apoptosis of thymocytes. Since the supernatant of the line had no significant effect on thymocyte death *in vitro* (data not shown) we hypothesized that the process depends on direct cell–cell contacts.

To check this hypothesis, we first incubated thymocytes with the TEC line at 4°C for 30 min to avoid transmission of apoptotic signals, removed non-adherent thymocytes and than further cultivated these cells for 6 and 24 h, respectively at 37°C with or without TEC. Results given in Fig. 3b shows statistically lower percentages of viable cells in cultures with R-TNC.1 cells compared to corresponding controls (adherent thymocytes cultivated without TEC), suggesting that our TEC line induces apoptosis of a subset of adherent thymocytes.

A number of TEC and TSC lines have been shown to induce apoptosis of thymocytes. The process is either mediated by soluble factors (Zilberman *et al.*, 1996; Rinner *et al.*, 1996) of which some possess glucocorticoid hormone activity (Zilberman *et al.*, 1996; Pazirandeh *et al.*, 1999) or by direct cell–cell contacts (Rinner *et al.*, 1994; Schreiber *et al.*, 1996; Zilberman *et al.*, 1999). A few studies examined the involvement of adhesion molecules in apoptosis of thymocytes in the contact with TEC lines. Schreiber *et al.* (1996) demonstrated that anti-CD2 and anti-LFA-1 mAbs inhibited apoptosis of mouse thymocytes triggered by TEC. Recently, it has been postulated that galectin-1, expressed on TEC (Baum *et al.*, 1995), could trigger apoptosis of both non-selected or negatively selected thymocytes (Perillo *et al.*, 1997).

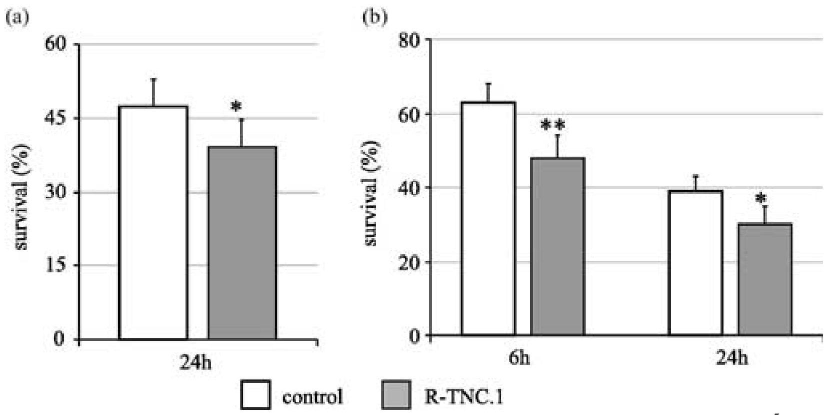

FIGURE 3 (a) Effect of the R-TNC.1 cell line on thymocyte survival in culture. Freshly isolated thymocytes ($5 \times 10^5$ cells/well) were cultivated with or without R-TNC.1 monolayers in 96-well plates for 24 h at 37°C as described. After that, thymocytes were collected by pipetting. TEC were carefully detached by scraping. The numbers of viable thymocytes (both free and intra-TNC) were calculated by tripan blue dye exclusion. Survival of thymocytes was determined as follows: number of viable thymocytes in culture/initial number of thymocytes $\times$ 100. Values are given as mean ± SD of triplicates of one out of three similar experiments. $*p < 0.05$ compared to corresponding control (thymocytes cultivated without TEC). (b) Effect of R-TNC.1 cell line on viability of adherent thymocytes in culture. Thymocytes were first incubated with R-TNC.1 cells at 4°C for 30 min. The unbound cells were removed and adherent thymocytes were further cultivated with or without R-TNC.1 monolayers for 6 and 24 h, respectively, as described. Viability was determined by tripan blue dye exclusion. Values are given as mean ± SD of triplicates of one out of three experiments. $*p < 0.05$; $**p < 0.01$ compared to control (adherent thymocytes cultivated without TEC).

### Dynamics of Thymocyte Emperiopolesis by the R-TNC.1 Cell Line

The nursing activity of the R-TNC.1 cell line was manifested by the engulfment (emperiopolesis) of thymocytes after their binding to TEC (Čolić *et al.*, 1994). The characteristics of the line appear to be identical with TNC reported by Wekerle *et al.* (1980), and mouse stromal cell lines which formed characteristics complex structures with thymocytes in the monolayers and hanging drop culture system (Itoh *et al.*, 1988; Nishimura *et al.*, 1990; Hiramine *et al.*, 1990; Hirata *et al.*, 1991; Li *et al.*, 1992). To our knowledge this is the first line with nursing characteristics established from the rat thymus.

In this work, we studied dynamics of emperiopolesis during 72 h. Results presented in Fig. 4 shows that internalization of thymocytes by R-TNC.1 cells was visible as early as 1.5 h of cell coculture and gradually increased until 24 h. After that, the number of engulfed thymocytes was significantly reduced.

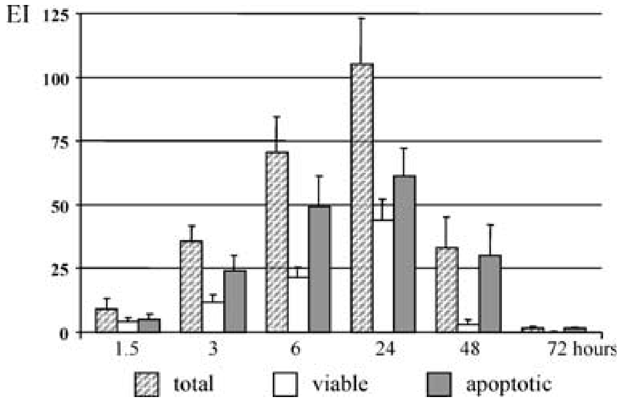

FIGURE 4 Dynamics of thymocyte emperiopolesis by the R-TNC.1 cell line. Thymocyte engulfment was determined as described after different coculture periods at 37°C. Viable and apoptotic thymocytes were distinguished based on morphological criteria. Values (mean ± SD of non-aplicates cultures) from one representative experiment (out of five different experiments with similar results) are given as engulfment index (EI = total number of internalized thymocytes/100 R-TNC.1 cells).

The dynamics of emperiopolesis was elegantly studied by Philp *et al.* (1993) using long term video microscopy. They demonstrated that movement of thymocytes through TNC is directionally controlled and that cytoplasmic channels within TNC became visible simultaneously with the initiation of internalization. They also detected several specialized contact structures between membranes of enclosed thymocytes and TNC. In our preliminary experiments, we found that emperiopolesis was much higher when R-TNC.1 cells were pretreated with mytomicin C, indicating that this procedure rearranges cytoskeletal filaments and enables either better formation of cytoplasmic channels or faster movement of intra-TNC thymocytes.

When the cells were fixed with formalin/ethanol a clear morphological distinction between viable and apoptotic cells among internalized thymocytes was observed (Fig. 5a–c). Apoptotic thymocytes were also identified using the TUNEL technique (Fig. 5d and e). Most engulfed thymocytes resided in common or individual vacuoles. Figure 4 shows that initially (1.5 h) almost equal percentage of apoptotic and viable thymocytes were seen inside the R-TNC.1 cells. After 6 and 24 h approximately 70 and 60% internalized thymocytes, respectively, were apoptotic. The percentages of apoptotic cells among engulfed thymocytes were much higher in all culture times examined than in corresponding controls (spontaneous apoptosis of thymocytes cultivated alone). Figures 5b and c also demonstrated that among viable thymocytes some are in mitosis as defined by the presence of mitotic figures. The number of both viable and apoptotic thymocytes among internalized cells significantly decreased when

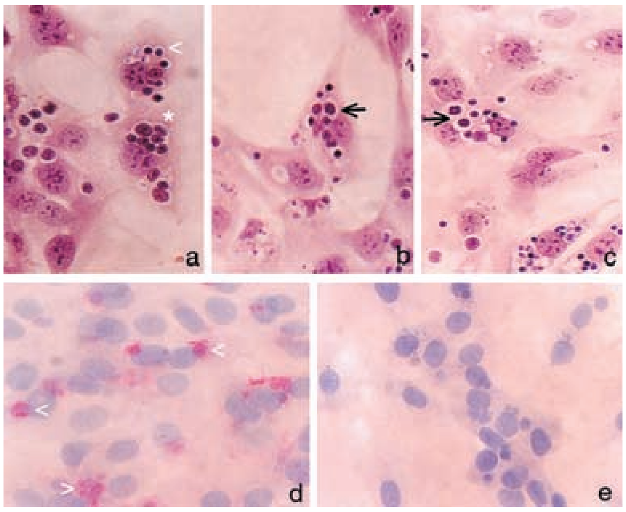

FIGURE 5 (a–c) Monolayers of R-TNC.1 cells with engulfed thymocytes after 24 h cultivation, fixed with 4% formalin in ethanol and stained with hematoxylin/eosin. Viable thymocytes are marked by white asterix, apoptotic thymocytes are marked with white arrowhead, whereas thymocytes with mitotic figures are indicated by black arrows. (d and e) Monolayers of R-TNC.1 cells with engulfed thymocytes after 24 h cultivation, stained by the TUNEL assay. Certain TUNEL$^+$ cells are indicated by white arrowheads (d). No staining was seen in control (e) in which TdT was omitted. Magnifications $\times$ 240.

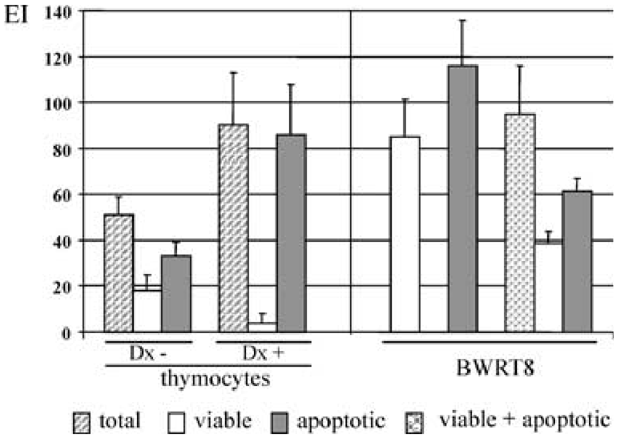

FIGURE 6 Emperiopolesis of viable and apoptotic thymocytes/TH by the R-TNC.1 cell line. Apoptosis was induced by cocultivation of thymocytes with 100 nM of Dx for 24 h or by prolonged TH cultivation (72 h) at higher density. Viable or Dx treated thymocytes, viable or apoptotic BWRT 8 cells as well as mixture of equal proportion of viable and apoptotic BWRT 8 cells were added to R-TNC.1 monolayers on multi spot glass slides. Engulfment was determined as described in "Materials and methods" section after 6 h of coculture at 37°C. The results are presented as mean ± SD of non-aplicates of one representative experiment out of three with similar results. EI = total numbers of internalized thymocytes(TH)/100 R-TNC.1 cells.

non-adherent thymocytes were removed after 6 h of cell coculture (data not shown).

In summary these results showed that the R-TNC.1 cell line internalizes both viable and apoptotic thymocytes and suggest different roles of this TEC line: phagocytosis; apoptosis induction of engulfed thymocytes; induction of proliferation of a subset of engulfed thymocytes. Neither of these studies regarding TNC-thymocyte interactions examined directly these processes. Therefore, this was our principal aim in creating the next experiments.

### Phagocytosis of Apoptotic Cells by the R-TNC.1 Cell Line

The phagocytic activity of TNC have been suggested by different authors on the basis of the presence of phagolysosomes with acid phosphatase activity into mouse TNC after cyclophosphamide treatment *in vivo* (Penninger *et al.*, 1994; Hiramine *et al.*, 1996) or into a TNC line *in vitro* (Hiramine *et al.*, 1990; Kawabuchi *et al.*, 1996). However, these experiments did not exclude the possibility that TNC internalize intact thymocytes and that apoptosis occurs inside TNC. Therefore we used a direct approach for measuring apoptosis (cocultivation of apoptotic cells with R-TNC.1 cells) and our term phagocytosis is restricted to ingestion of dead cells.

In these experiments, apoptotic, Dx treated thymocytes or apoptotic BWRT 8 cells (died due to deprivation of nutrition factors) were used. Figure 6 shows that the R-TNC.1 line efficiently internalizes both types of apoptotic cells. Phagocytosis was followed by intracellular destruction of apoptotic cells. As a consequence of the process many vacuoles with cellular debris appeared. Figure 6 also shows that internalization of apoptotic cells by R-TNC.1 cell line was higher than viable cells.

These findings together with previous observation (Aguilar *et al.*, 1994; Hiramine *et al.*, 1996) suggest that TNC could function like thymic macrophages in removing apoptotic cells. The process is of importance when massive thymocytes apoptosis occurs in the thymus after treatment with Dx, cyclophosphamide, X-ray irradiation or anti-CD3 antibody (Leene *et al.*, 1988; Čolić and Lilić 1991; Aguilar *et al.*, 1994; Hiramine *et al.*, 1996). A large number of TNC-like structures containing many apoptotic cells that appear under such experimental conditions (Leene *et al.*, 1988; Hiramine *et al.*, 1996) could be a consequence of programmed cell death of intra-TNC thymocytes but also due to phagocytosis of thymocytes died outside TNC. Physiologically, phagocyte activity of TNC could be relevant for removing non-selected or negatively selected thymocytes.

### Intra-TNC Apoptosis of Engulfed Viable Thymocytes

To check whether R-TNC.1 cell line induces apoptosis of internalized thymocytes we cocultivated thymocytes with the TEC line for 6 or 24 h, respectively, removed non-adherent and adherent thymocytes and left TEC with internalized thymocytes for additional 24 h in culture (second cultivation). After that the number of viable internalized thymocytes was calculated and expressed as the percentage of cell survival compared to the number of

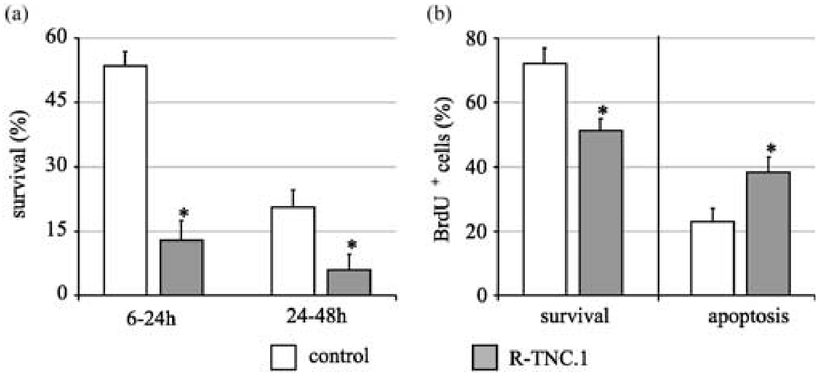

FIGURE 7 Effect of R-TNC.1 cells on survival and apoptosis of internalized thymocytes. (a) R-TNC.1 cells were incubated with thymocytes at 37°C for 6 or 24 h on glass multi spot slides (first cultivation step). After removal of unbound and bound thymocytes, TEC with engulfed thymocytes were further cultivated for 24 h (second cultivation step). Viable and apoptotic thymocytes were distinguished based on morphological criteria. The number of viable internalized thymocytes was calculated after second cultivation step and expressed as the percentage of cell survival compared to the number of viable intra-TNC thymocytes after the first cultivation step. Values are given as mean ± SD of sixplicates cultures from one representative experiment out of three experiments with similar results. *$p < 0.001$ compared to corresponding controls (survival of thymocytes cultivated without TEC). (b) Thymocytes were incubated with BrdU (10 μM) *in vitro* for 4 h. After that they were cultivated alone or with R-TNC.1 cells. After 24 h (first cultivation step) slides were thoroughly washed and R-TNC.1 with internalized thymocytes was cultivated for additional 24 h (second cultivation step). Results are presented as the percentage of survival or apoptosis of $BrdU^+$ intra-TNC thymocytes in comparison to the number of BrdU + cells with intact or apoptotic nuclear morphology engulfed by 1000 R-TNC.1 cells after first cultivation step. Values are mean ± SD of 4-plicates cultures from one representative experiment out of three experiments with similar results. *$p < 0.001$ compared to survival or apoptosis of $BrdU^+$ thymocytes cultivated without TEC (control).

viable internalized thymocytes before the second cultivation step. The comparison seems to be valid since our preliminary experiments showed that during the second cultivation step most intra-TNC thymocytes remained on site and that their release into culture medium was lower than 0.1%. Results of survival of intra-TNC thymocytes were compared to those of corresponding controls (thymocytes cultivated without TEC). As shown in Fig. 7a survival of engulfed intra-TNC thymocytes was much lower than survival of whole thymocytes suggesting that the R-TNC.1 cell line might induce apoptosis of intra-TNC thymocytes. However, these results did not exclude the possibility that among ingested viable cells some are already triggered to die outside TNC before internalization. Therefore one approach to explore this problem was to use 5-bromo-2′deoxyuridine (BrdU) labeled thymocytes, which represents a subset of proliferating cells. Results given in Fig. 7b confirmed the previous ones that survival of intra-TNC $BrdU^+$ cells was lower than survival of control $BrdU^+$ cells (cultivated without TEC) which was a consequence of higher apoptosis of intra-TNC $BrdU^+$ cells.

Cumulatively our results directly demonstrated that the R-TNC.1 cell line is able to induce apoptosis of a subset of both bound and internalized thymocytes. The phenomenon could be relevant for selection processes *in vivo*. If a developing thymocyte in close contact with TNC did not receive a positively selecting signal TNC could trigger a signal leading the thymocyte to apoptosis by neglect. Alternatively, it can be postulated that TNC might have a role in negative selection of thymocytes that express high affinity TCR for the complex self MHC/self peptide. TEC line mediating both positive and negative selection has been already described (Vukmanovic *et al.*, 1994).

### R-TNC.1 Cell Line Stimulates Proliferation of Engulfed Thymocytes and Thymocyte Hybridomas

Initial experiments (Fig. 5b and c) demonstrated that among engulfed thymocytes some are in mitosis. Similar observation have been published for TNC *in situ* (Wekerle *et al.*, 1980; Brelinska and Warchol, 1997) and TNC lines *in vitro* (Hiramine *et al.*, 1990; Nishimura *et al.*, 1990). Therefore to explore this phenomenon in more details we determined the percentage of mitotic intra-TNC at 6 and 24 h, respectively. Results presented in Fig. 8 shows that after 24 h, the percentage of mitotic intra-TNC thymocytes was statistically higher than the values for control cells (thymocytes in mitosis in cultures without TEC). Higher proliferation of a subset of engulfed thymocytes in comparison to control was also seen 6 and 24 h in culture when proliferation was measured by BrdU incorporation (Figs. 8 and 9a,b).

The R-TNC.1 cell line also significantly stimulated proliferation of engulfed BWRT 8 cells as judged by both identification of mitotic figures or BrdU incorporation (Figs. 8 and 9c,d). As expected the level of spontaneous proliferation of BWRT 8 cells was much higher in comparison to thymocytes. These results suggest that TNC provide specific microenvironment for T cell development. It is known that the process of thymic education in which thymocytes learn to distinguish between self and foreign antigens occurs at the double positive (DP) stage of development and involves interaction between TCR and coreceptor molecules on

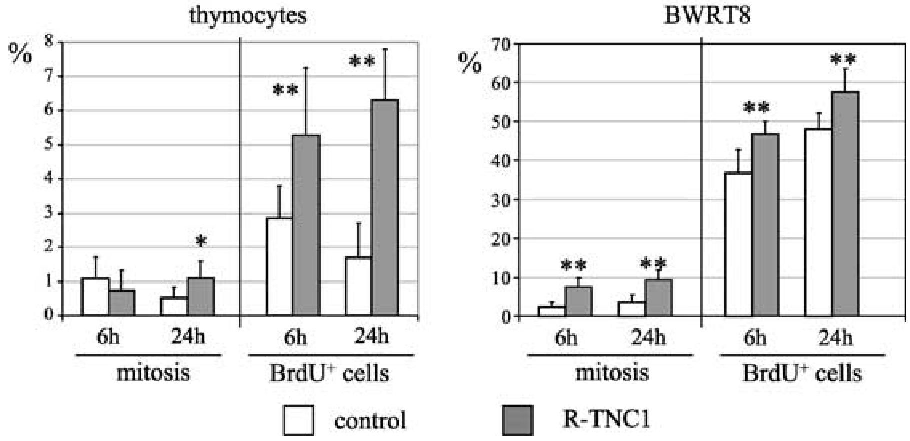

FIGURE 8 Effect of the R-TNC.1 cell line on proliferation of intra-TNC thymocytes (a) and TH (b). Proliferation was determined by morphological criteria (presence of mitotic figures) and by BrdU incorporation as described in “Materials and methods” section. Results are presented as the percentage of mitotic thymocytes (TH) or $BrdU^+$ thymocytes (TH) engulfed by R-TNC.1 cells after 6 and 24 h of cocultivation, respectively, compared to corresponding controls (thymocytes/TH cultivated alone). Values are mean ± SD of non-aplicates from one representative experiment out of at least five experiments with similar results. $*p < 0.05$, $**p < 0.01$ compared to control (thymocytes cultivated without TEC).

thymocytes and MHC/self antigens on thymic stromal cells (Anderson *et al.*, 1996). TNC express class I and II MHC antigens on their plasma membranes as well as on the membranes of specialized cytoplasmic vacuoles (Wekerle *et al.*, 1980; de Waal Malefijt *et al.*, 1986; Ezaki and Uehara, 1997). As mentioned before TNC also contain some endosomal and lysosomal machinery which makes them capable for antigen presentation and antigen processing (Penninger *et al.*, 1994).

In the context of the role of TNC in positive selections of thymocytes several earlier reports indicated that murine TNC lines support the growth and differentiation of DP thymocytes (Nishimura *et al.*, 1990; Gao *et al.*, 1993), maintain or increase the viability of a subset of intra-TNC thymocytes (Pezzano *et al.*, 1995; 1996). de Waal Malefijt *et al.* (1986) showed that a minority of intra-TNC thymocytes survives Dx treatment *in vivo* and suggested that differentiation of thymocytes from DP cells to cortisone-resistant single positive (SP) occurs within TNC. Hiramine *et al.* (1996) also found fully intact thymocytes within TNC after cyclophosphamide treatment, suggesting that TNC participate in the nursing of immature thymocytes and selection processes. Brelinska (1989) showed that intra TNC thymocytes *in vivo* underwent DNA synthesis and enter mitosis indicating that microenvironmental factors within TNC could induce thymocytes to enter cell cycle. In these processes various soluble factors produced by the TNC epithelium such as thymic hormones, neurohypophysial related peptides and cytokines (IL-6, IL-7, IL-8, IL-9, G-CSF, GM-CSF, CSF-1, TGF-β, c-kit ligand) might be involved (Geenen *et al.*, 1992; 1993; Deman *et al.*, 1994; Iwagami *et al.*, 1994).

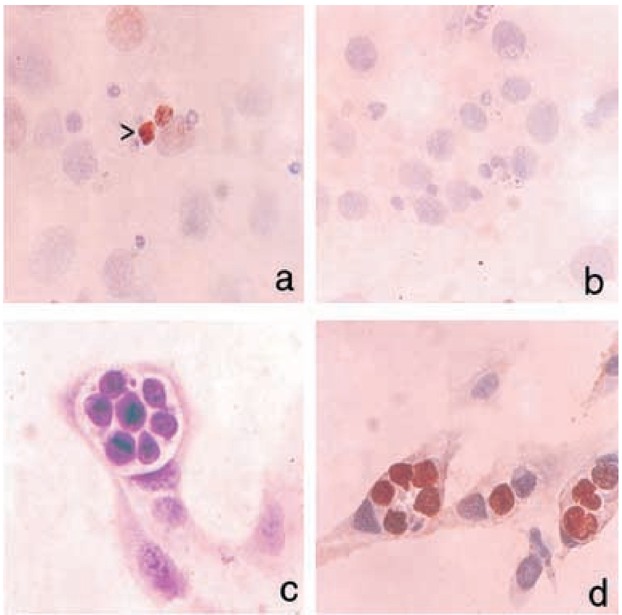

FIGURE 9 Intra-TNC proliferation of thymocytes and TH cells. (a) Monolayers of R-TNC.1 cells with engulfed thymocytes after 6 h cultivation stained with anti-BrdU antibody. Positive cells were indicated by black arrowhead. (b) Negative control (primary antibody omitted). (c) Monolayers of R-TNC.1 cells with engulfed BWRT 8 hybridoma stained with hematoxylin/eosin. Note mitotic figures. (d) Intra-TNC TH stained with anti-BrdU antibody. Most cells are $BrdU^+$. Magnification × 240.

### Concluding Remarks

Taken together, our results demonstrate the ability of R-TNC.1 cell line to bind and engulf viable and apoptotic thymocytes and TH cells, to induce apoptosis of both adherent and internalized thymocytes and to stimulate proliferation of a subset of intra-TNC thymocytes or TH cells. These data suggest that a single TNC, by providing specific microenvironments, might have different roles in T-cell development: phagocytic activity; nursing activity; capability to stimulate proliferation of thymic T cells and probably to mediate selection processes. Therefore, the line is an excellent tool for studying these processes *in vitro*.

### MATERIALS AND METHODS

### Animals

AO rats, both sexes, 8–10 weeks old, bred at the Farm for Experimental Animals (MMA, Belgrade) were used in this study.

### Cells

The R-TNC.1 cell line was established at the Institute of Medical Research, MMA, Belgrade from a long-term rat thymic culture as previously described (Čolić *et al.*, 1994). TEC were cultivated in RPMI 1640 medium containing 15% fetal calf serum (FCS), 5 μg/ml insulin, 50 nM Dx, 10 ng/ml epidermal growth factor. All chemicals were obtained from Sigma, Munich, Germany.

BWRT 8 TH was selected from a fusion of the mouse thymoma cell line BW5147 and ConA + IL-2 activated rat thymocytes (Popović *et al.*, 1994).

Thymocytes were prepared by teasing thymuses through a stainless steel mesh. Large aggregates were removed and the cells were washed with RPMI medium + 5% FCS before use. The viability assessed by trypan blue staining was usually greater than 95%. All cultures were maintained at 37°C in a humidified atmosphere containing 5% $CO_2$.

Apoptotic thymocytes were prepared by treatment of the cells by Dx (100 nM) (Sigma) for 24 h. Apoptosis of TH was induced by prolonged cultivation (72 h) at higher density ($5 \times 10^5$ cells/ml).

### Binding Assay

R-TNC.1 cells were trypsinized by 0.1% trypsin in 0.02% EDTA/PBS and plated in 96-well flat bottomed plates ($7 \times 10^3$ cells/well in 200 μl of medium) or on multi spot slides ($3 \times 10^3$ cells/well in 50 μl of medium) and cultivated in TEC culture medium until confluent monolayers were obtained usually 2–3 days. In the coculture assay enriched medium used for standard cultivation of these cells was replaced with RPMI medium containing 10% FCS. Resting adult thymocytes ($5 \times 10^5$ cells/well) or BWRT 8 TH ($5 \times 10^4$ cells/well) were added onto confluent monolayers. After incubation for various period of time at 37°C the plates were spun upside down (100 g 30 s) and quickly filled with medium to prevent cell drying. Thymocytes were detached from monolayers by pipetting. The number of adherent cells was counted and percentage of binding was calculated as follows: %binding = (number of adherent thymocytes or TH/number of total thymocytes or TH) × 100.

In experiments performed on 1 × 10 well multi spot glass slides (ICN, Costa Mesa, CA) thymocytes ($1 \times 10^5$/spot) were cultivated with confluent TEC monolayers. Unattached thymocytes were removed by multiple washing with phosphate buffered saline (PBS). After fixation (4% formaldehyde in ethanol) and washing in distilled water slides were stained with hematoxylin–eosin, and analyzed under a light microscope. The number of adherent thymocytes per 500 R-TNC.1 cells was counted on each spot and presented as adhesion index (number of bound thymocytes/100 R-TNC.1 cells).

### Engulfment Assay

R.TNC.1 cells were plated onto multi spot glass slides ($3 \times 10^3$ cells/spot) overnight in complete RPMI medium with 15% FCS. The next day, the medium was replaced with RPMI medium with 10% FCS and thymocytes ($5 \times 10^5$) or TH ($5 \times 10^4$) were added to each spot and incubated for different periods (1–72 h) at 37°C. The slides were placed into 4 well plates (one slide/well) and carefully covered with medium. After cultivation the slides were vigorously washed in PBS to remove all non-adherent and adherent thymocytes or TH, fixed in 4% formaldehyde and stained with hematoxylin–eosin. The number of internalized thymocytes (TH) by 500 R-TNC.1 cells was counted on each spot under a light microscope.

The degree of internalization was presented as engulfment index (EI). EI = total number of internalized thymocytes or TH/100 R-TNC.1 cells. In some experiments results were expressed as the percentage of relative engulfment compared to control values used as 100%.

In experiments in which the fate of internalized thymocytes (TH) was studied after prolonged cultivation (24–72 h), R-TNC.1 cells were treated with mytomicin C (25 μg/ml) for 30 min at 37°C, to prevent the overgrowth of epithelial cells.

### Apoptosis Assay

Apoptosis was determined by morphological analysis and TUNEL assay. For morphological analysis cells were fixed in 4% formaldehyde in ethanol overnight at 4°C. After washing in distilled water, cells were stained with hematoxylin–eosin and analyzed under light microscope. Apoptotic cells were defined based on well known morphological characteristics: chromatin condensation, nuclear pyknosis and nuclear fragmentation.

The TUNEL assay was performed using the "*In Situ* Cell Death detection Kit, AP" (Boehringer Mainheim, Germany). Air dried monolayers of R-TNC.1 cells with engulfed thymocytes were washed with PBS and fixed with paraformaldehyde solution (4% in PBS pH 7.4) for 30 min at room temperature. After washing with PBS slides were incubated in permeabilization solution (0.1% triton-X in 0.1% sodium citrate) for 2 min on ice (4°C). After that, slides were rinsed twice with PBS and incubated with the TUNEL reaction mixture (terminal deoxynucleotidyl transferaze and nucleotide mixture labeled with FITC) in a humidified chamber for 60 min at 37°C. Rinsed slides were than incubated with converter

AP (anti fluorescein antibody, conjugated with alkaline phosphatase) under the same condition for 30 min. Finally, the slides were incubated with substrate solution for 10 min at room temperature and after rinsing with PBS analyzed by a light microscope.

In experiments in which apoptosis of intra-TNC thymocytes was examined, thymocytes were firstly incubated with a thymidine analogue, BrdU (10 μM) (Biochemical Corporation Cleveland, OH, USA) *in vitro* for 4 h and than cultivated alone in 96-well plates or with R-TNC.1 cells on multi spot glass slides. After 24 h (first cultivation step) slides were thoroughly washed. A part of washed slides was fixed with 4% formalin/ethanol, whereas the rest was cultivated for additional 24 h (second cultivation step). Similar procedure was done for thymocytes cultivated without TEC. From these cells cytospins were prepared using a cytocentrifuge.

Fixed slides and cytospins were treated with Tween 20 0.5 + 0.2%BSA for 15 min to permeabilize cells. After washing in PBS, the slides were treated with 4 N HCl and 0.1 M $Na_2B_4O_7$ for 20 and 5 min, respectively. Slides were than washed in PBS and incubated with anti-BrdU antibody (Serva, Heidelberg, Germany) (1:30). Detection of bound antibody was achieved using a peroxidase conjugated secondary antibody to mouse Ig (DAKO, Denmark) and diaminobenzidine (DAB) (Serva, Heidelberg, Germany) and 0.01% $H_2O_2$ as substrate.

Based on nuclear morphology of $BrdU^+$ cells apoptotic and non-apoptotic $BrdU^+$ thymocytes were identified and calculated. Results were expressed as the percentage of survival of $BrdU^+$ intra-TNC thymocytes by comparing the number of $BrdU^+$ cells with intact nuclear morphology internalized by 1000 R-TNC.1 cells on each spot after second cultivation step, with the number of $BrdU^+$, viable intra-TNC cells after the first cultivation step calculated at the same manner. Results are also expressed as percentage of apoptotic $BrdU^+$ cells determined on the basis of total number of intra-TNC $BrdU^+$ cells engulfed by 1000 R-TNC.1 cells.

### Intra-TNC Proliferation Assay

Proliferation rate of thymocyte/TH inside R-TNC.1 cells was measured by morphological analysis (calculation of TH with mitotic figures inside R-TNC.1 cells) as well as using BrdU incorporation. For morphological analysis the slides were prepared at the same way as for morphological determination of apoptosis. In experiments in which proliferation is determined by BrdU, the compound (10 μM), was added in cultures of R-TNC.1/thymocytes (TH) 30 min before termination of appropriate incubation period. After that, monolayers were washed in PBS and dried overnight. The slides were than fixed in acetone (10–15 min at room temperature) and proceeded for immunoperoxidase staining using anti-BrdU mAb as previously described.

### Acknowledgements

The authors thank Petar Milosavljevic for excellent technical assistance.

### References

Aguilar, L.K., Aguilar-Cordova, E., Cartwright, Jr., J. and Belmont, J.W. (1994) "Thymic nurse cells are sites of thymocyte apoptosis", *J. Immunol.* **152**, 2645–2651.

Anderson, G., Moore, N.C., Owen, J.J.T. and Jenkinson, E.J. (1996) "Cellular interactions in thymocyte development", *Annu. Rev. Immunol.* **14**, 73–99.

Baum, L.G., Pang, M., Perillo, N.L., Wu, T., Delegeane, A., Uittenbogaart, C.H., Fukuda, M. and Seilhamer, J.J. (1995) "Human thymic epithelial cells express endogenous lectin, galectin-1, which binds to core 2 O-glycans on thymocytes and T lymphoblastoid cells", *J. Exp. Med.* **181**, 877–887.

Brelinska, R. (1989) "Thymic nurse cells: division of thymocytes within complexes", *Cell Tissue Res.* **258**, 637–643.

Brelinska, R. and Warchol, J.B. (1997) "Thymic nurse cells: Their functional ultrastructure", *Microsc. Res. Tech.* **38**, 250–266.

Čolić, M. and Lilić, D. (1991) "The effect of trauma on the thymus", In: Kendal, M.D. and Ritter, M.A., eds, *Thymus Update 4. The Thymus in Immunotoxicology* (Harwood Academic Publishers, London), pp. 3–34.

Čolić, M., Vučević, D., Miyasaka, M., Tamatani, T., Pavlović, M.D. and Dujić, A. (1994) "Adhesion molecules involved in the binding and subsequent engulfment of thymocytes by a rat thymic epithelial cell line", *Immunology* **83**, 449–456.

Čolić, M., Vučević, D., Pavlović, M.D., Lukić, T., Milinković, M., Popović, L.J., Popović, P. and Dujić, A. (1997) "Adhesion molecules in the thymic microenvironment: interaction between thymocytes and cloned thymic epithelial cell lines", In: Lukić, M.L., Čolić, M., Mostarica-Stojković, M. and Čuperlović, K., eds, *Immunoregulation in Health and Disease. Experimental and Clinical Aspects* (Academic Press, London), pp. 13–33.

Čolić, M., Vučević, D., Popović, P. and Dujić, A. (1998) "Bidirectional interactions between thymocytes and thymic epithelial cell lines *in vitro*", *Dev. Immunol.* **6**, 71–79.

Deman, J., Humblet, C., Martin, M.T., Boniver, J. and Defresne, M.P. (1994) "Analysis by *in situ* hybridization of cytokine mRNAS expression in thymic nurse cells", In: Heinen, E., Defresne, M.P., Boniver, J. and Geenen, V., eds, *In vivo Immunology. Regulatory Processes During Lymphopoiesis and Immunopoiesis* (Plenum Press, New York).

Ezaki, T. and Uehara, Y. (1997) "Thymic nurse cells forming a dynamic microenvironment in spontaneous thymoma BUF/Mna rats", *Arch. Histol. Cytol.* **60**, 39–51.

Ezaki, T., Matsuno, K. and Kotani, M. (1991) "Thymic nurse cells (TNC) in spontaneous thymoma BUF/Mna rats as a model to study their roles in T-cell development", *Immunology* **73**, 151–158.

Gao, X., Nishimura, T., Takeuchi, Y., Sudo, T. and Habu, S. (1993) "Thymic nurse cell clone supports the differentiation of CD4 − 8 − thymocytes into CD4 + 8 + thymocytes *in vitro*", *Immunol. Lett.* **35**, 169–175.

Geenen, V., Martens, H., Robert, F., Vrindts-Gevaert, Y., De-Groote, D. and Franchimont, P. (1992) "Immunomodulatory properties of cyclic hexapeptide oxytocin antagonists", *Thymus* **20**, 217–226.

Geenen, V., Martens, H., Corman-Goffin, N., Vandersmissen, E., Legros, J.J., De Groote, D., Defresne, M.P., Boniver, J. and Franchimont, P. (1993) "Central function of the thymus in the recognition of neuroendocrine functions by T Lymphocytes during their development", *Arch. Int. Physiol. Biochem. Biophys.* **101**, 19–22.

Hiramine, C., Hojo, K., Koseto, M., Nakagawa, T. and Mukasa, A. (1990) "Establishment of a murine thymic epithelial cell line capable of inducing both thymic nurse cell formation and thymocyte apoptosis", *Lab. Investig.* **62**, 41–53.

Hiramine, C., Nakagawa, T. and Hojo, K. (1995) "Murine nursing thymic epithelial cell lines capable of inducing thymocyte apoptosis express the self-superantigen Mls-1a", *Cell Immunol.* **160**, 157–162.

Hiramine, C., Nakagawa, T., Miyauchi, A. and Hojo, K. (1996) "Thymic nurse cells as the site of thymocyte apoptosis and apoptotic cell clearance in the thymus of cyclophosphamide-treated mice", *Lab. Investig.* **75**, 185–201.

Hirata, K., Mori, K., Nakamura, K., Kawabuchi, M. and Watanabe, T. (1991) "Morphological analysis of the cellular interaction between thymocytes and a thymic stromal cell line (TEL-2)", *Anat. Rec.* **230**, 524–530.

Itoh, T., Doi, H., Chin, S., Nishimura, T. and Kasahara, S. (1988) "Establishment of mouse thymic nurse cell clones from a spontaneous BALB/c thymic tumor", *Eur. J. Immunol.* **18**, 821–824.

Iwagami, S., Furue, S., Toyosaki, T., Horikawa, T., Doi, H., Satomi, S., Itoh, T., Sakata, T. and Suzuki, R. (1994) "Establishment and characterization of nurse cell like clones from human skin. Nurse cell-like clones can stimulate autologous mixed lymphocyte", *J. Immunol.* **153**, 2927–2938.

Kawabuchi, M., Nakamura, K., Hirata, K., Mori, K., Kishi, H., Islam, S., Chongjian, Z. and Watanabe, T. (1996) "Morphological study of thymus stromal cells (TEL-2 cell) which play a role in the elimination of double positive immature thymocytes by phagocytosis", *Anat. Rec.* **244**, 271–283.

Kishimoto, H., Surch, C.D. and Sprent, J. (1995) "Upregulation of surface markers on dying thymocytes", *J. Exp. Med.* **181**, 649–655.

Kyewski, B.A. (1986) "Thymic nurse cells: possible sites of T-cell selection", *Immunol. Today* **7**, 374–379.

Leene, W., de Waal Malefijt, R., Roholl, P.J.M. and Hoeben, K.A. (1988) "Lymphocyte depletion in thymic nurse cells: a tool to identify *in situ* lympho-epithelial complexes having thymic nurse cell characteristics", *Cell Tissue Res.* **253**, 61–68.

Lepesant, H., Reggio, H., Pierres, M. and Naquet, P. (1990) "Mouse thymic epithelial cell lines interact with and select a CD3lowCD4 + CD8 + thymocyte subset through an LFA-1-dependent adhesion—de-adhesion mechanism", *Int. Immunol.* **2**, 1021–1031.

Li, Y., Pezzano, M., Philp, D., Reid, V. and Guyden, J. (1992) "Thymic nurse cells exclusively bind and internalize CD4 + CD8 + thymocytes", *Cell Immunol.* **140**, 495–506.

Mukamoto, M., Okada, T., Kodama, H. and Baba, T. (1999) "Effects of chicken thymic stromal cells on the growth and differentiation of thymocytes *in vitro*", *Vet. Immunol. Immunopathol.* **68**, 25–37.

Nishimura, T., Takeuchi, Y., Ichimura, Y., Gao, X., Akatsuka, A., Tamaoki, N., Yagiota, H., Okumura, K. and Habu, S. (1990) "Thymic stromal cell clone with nursing activity supports the growth and differentiation of murine CD4 + 8 + thymocyte *in vitro*", *J. Immunol.* **145**, 4012–4017.

Oliveira-dos-Santos, A.J., Penninger, J.M., Rieker-Geley, T., Matsumoto, G., Mak, T.M. and Wick, G. (1998) "Thymic heterotypic cellular complexes in gene-targeted mice with defined blocks in T cell development and adhesion molecule expression", *Eur. J. Immunol.* **28**, 2882–2892.

Pazirandeh, A., Xue, Y., Rafter, I., Sjovall, J., Jondal, M. and Okret, S. (1999) "Paracrine glucocorticoid activity produced by mouse thymic epithelial cells", *FASEB* **13**, 893–901.

Penninger, J., Rieker, T., Romani, N., Klima, J., Salvenmose, W., Dietrich, H., Stossei, H. and Wick, G. (1994) "Ultrastructural analysis of thymic nurse cell epithelium", *Eur. J. Immunol.* **24**, 222–228.

Perillo, N.L., Uittenbogaart, C.H., Nguyen, J.T. and Baum, L.G. (1997) "Galectin-1, an endogenous lectin produced by thymic epithelial cells, induces apoptosis of human thymocytes", *J. Exp. Med.* **185**, 1851–1858.

Pezzano, M., Li, Y., Philp, D., Omene, C., Cantey, M., Saunders, G. and Guyden, J. (1995) "Thymic nurse cell rescue of early CD4 + CD8 + thymocytes from apoptosis", *Cell Mol. Biol.* **41**, 1099–1111.

Pezzano, M., Philp, D., Stephenson, S., Li, Y., Reid, V., Maitta, R. and Guyden, J. (1996) "Positive selection by thymic nurse cells requires IL-1β and is associated with an increased Bcl-2 expression", *Cell Immunol.* **169**, 174–184.

Philp, D., Pezzano, M., Li, Y., Omene, C., Boto, W. and Guyden, J. (1993) "The binding, internalization and release of thymocytes by thymic nurse cells", *Cell Immunol.* **148**, 301–315.

Popović, L.J., Čolić, M. and Kosec, D. (1994) "Production and characterization of the rat thymic T-cell hybridomas", *Vojnosanit. Pregl.* **51**, 56–59.

Rieker, T., Penninger, J., Romani, N. and Wick, G. (1995) "Chicken thymic nurse cells: an overview", *Dev. Comp. Immunol.* **19**, 281–289.

Rinner, I., Kukulansky, T., Felsner, P., Skreiner, E., Globerson, A., Kasai, M., Hirokawa, K., Korsatko, W. and Schauenstein, K. (1994) "Cholinergic stimulation modulates apoptosis and differentiation of murine thymocytes via a nicotinic effect on thymic epithelium", *Biochem. Biophys. Res. Commun.* **203**, 1057–1062.

Rinner, I., Eren, R., Skreiner, E., Kukulansky, T., Kassai, M., Hirokawa, K., Globerson, A. and Schauenstein, K. (1996) "Thymocyte-directed enhancement of apoptosis via soluble factor(s) derived from a cortical and a medullary thymic epithelial cell line", *Cell Tissue Res.* **284**, 327–330.

Schreiber, L., Sharabi, Y., Schwartz, D., Goldfinger, N., Brodie, C., Rotter, V. and Shoham, J. (1996) "Induction of apoptosis and p53 expression in immature thymocytes by direct interaction with thymic epithelial cells", *Scand. J. Immunol.* **44**, 314–322.

Villa-Verde, D.M.S., Lagrota-Candido, J.M., Vannier-Santos, M.A., Chammas, R., Brentani, R.B. and Savino, W. (1994) "Extracellular matrix components of the mouse thymus microenvironment IV. Modulation of thymic nurse cells by extracellular matrix ligands and receptors", *Eur. J. Immunol.* **24**, 659–664.

Villa-Verde, D.M.S., Mello-Coelho, V., Lagrota-Candido, J.M., Chammas, R. and Savino, W. (1995) "The thymic nurse cell complex: an *in vitro* model for extracellular matrix-mediated intrathymic T cell migration", *Braz. J. Med. Biol. Res.* **28**, 907–912.

Vukmanović, S., Jameson, S.C. and Bevan, M. (1994) "A thymic epithelial cell line induces both positive and negative selection in the thymus", *Int. Immunol.* **5**, 239–246.

de Waal Malefijt, R., Leene, W., Roholl, P.J.M., Wormmeester, J. and Hoeben, K.A. (1986) "T cell differentiation within thymic nurse cells", *Lab. Investig.* **55**, 25–34.

Wekerle, H., Ketelsen, U.P. and Ernst, M. (1980) "Thymic nurse cells. Lymphoepithelial cell complexes in murine thymuses: Morphological and serological characterization", *J. Exp. Med.* **151**, 925–944.

Zilberman, Y., Yefenof, E., Oron, E., Dorogin, A. and Guy, R. (1996) "T cell receptor-independent apoptosis of thymocyte clones induced by a thymic epithelial cell line is mediated by steroids", *Cell Immunol.* **25**, 78–84.

Zilberman, Y., Yefenof, E., Katzav, S., Dorogin, A., Rosenheimer-Goudsmid, N. and Guy, R. (1999) "Apoptosis of thymic lymphoma clones by thymic epithelial cells: a putative model for 'death by neglect'", *Immunol. Lett.* **67**, 95–104.

